# Incidence and associated factors of delirium after primary total joint arthroplasty in elderly patients: A systematic review and meta-analysis

**DOI:** 10.1097/MD.0000000000038395

**Published:** 2024-05-31

**Authors:** Yanju Zhang, Yanjie Yu, Ziyu Han, Li Diao, Runping Zhao, Jinzhu Liu, Yuhong Luo, Huiyuan Wu, Yanjiang Yang

**Affiliations:** a Nursing Department, Cangzhou People’s Hospital, Cangzhou, Hebei, People’s Republic of China; b Hebei Medical University, Shijiazhuang, Hebei, People’s Republic of China; c Pharmacy Department, Cangzhou People’s Hospital, Cangzhou, Hebei, People’s Republic of China; d Oncology Department, Cangzhou People’s Hospital, Cangzhou, Hebei, People’s Republic of China; e Trauma Emergency Center, The Third Hospital of Hebei Medical University, Shijiazhuang, Hebei, People’s Republic of China.

**Keywords:** delirium, elderly patients, primary total joint arthroplasty, risk factors

## Abstract

**Background:**

A total of 1.5% to 20.2% of total joint arthroplasty patients experience delirium. Until now, no formal systematic review or meta-analysis was performed to summarize the risk factors of delirium after primary total joint arthroplasty (TJA).

**Methods:**

A comprehensive search encompassing Medline, Embase, and the Cochrane central database was conducted, incorporating studies available up to June 2023. We systematically reviewed research on the risk factors contributing to delirium following TJA in elderly patients, without language restrictions. The methodological quality of the included studies was evaluated using the Newcastle–Ottawa Scale. Data synthesis through pooling and a meta-analysis were performed to analyze the findings.

**Results:**

A total of 23 studies altogether included 71,095 patients with primary TJA, 2142 cases of delirium occurred after surgery, suggesting the accumulated incidence of 3.0%. The results indicated that age, current smoker, heavy drinker, mini-mental state examination score, hypertension, diabetes mellitus, chronic kidney disease, history of stroke, coronary arterial disease, dementia, history of psychiatric illness, American Society of Anesthesiologists physical status III–IV, general anesthesia, anesthesia time, operative time, intraoperative blood loss, blood transfusion, β-blockers, ACEI drugs, use of psychotropic drugs, preoperative C-reactive protein level, and preoperative albumin level were significantly associated with postoperative delirium after primary TJA.

**Conclusions:**

Related prophylaxis strategies should be implemented in the elderly involved with above-mentioned risk factors to prevent delirium after primary TJA.

## 1. Introduction

Postoperative delirium (POD) is a frequent and severe complication in elderly patients undergoing total joint arthroplasty (TJA).^[1]^ Previous studies have demonstrated that delirium is associated with a 4-fold increase in mortality,^[2]^ a 2.4-fold increase in healthcare costs,^[3]^ prolonged hospitalization and worse functional recovery.^[4]^ The etiology of delirium is unclear, and prevention remains the most cost-effective strategy. Previous studies have mainly focused on identifying risk factors for primary prevention of delirium.

Several individual studies^[5–8]^ have explored and confirmed various risk factors for postoperative delirium after TJA, including age, sex, comorbidities, medications, anesthesia type, blood transfusion, etc. However, these results are limited by heterogeneity, confounding, bias, and small sample size. For example, the association between blood transfusion and POD is contradictory among different studies.^[9,10]^ The incidence of POD also varies widely depending on the characteristics of the study population, the design of the study, the methods of assessing delirium, the follow-up period, and other factors.^[1,8]^ These inconsistent or highly variable results make it difficult for clinicians to have a clear and comprehensive understanding of the risk factors for this adverse event, and to implement some preventive interventions. Therefore, it is necessary to conduct a systematic review to quantify the contribution effects of these factors.

Until now, no formal systematic review or meta-analysis was performed to summarize the risk factors of delirium after primary TJA. Consequently, our study undertook a meta-analysis to amalgamate the existing evidence and ascertain the risk factors underpinning postoperative delirium after TJA.

## 2. Materials and methods

This systematic review and meta-analysis adhered to the guidelines stipulated by the Preferred Reporting Items for Systematic Reviews and Meta-Analyses (PRISMA) Statement. Additionally, the study’s registration was completed at the International Prospective Register of Systematic Reviews (number CRD42021254606).

### 2.1. Literature search

Medline, Embase, and Cochrane central database were searched using a broad range of terms to identify original research, published all through June 2023 and selecting potential studies to consider. The main key words were as follows: “predictor” or “risk” or “factor” AND “delirium” AND “total joint arthroplasty” or “ total hip and knee arthroplasty” AND “elderly.” Also, a manual search of references in the identified articles and systematic reviews was performed for possible inclusion.

### 2.2. Eligibility criteria

Two reviewers (Y.Z. and Y.Y.) independently assessed the titles and abstracts of the identified studies. Only full-text articles without language restrictions were considered for inclusion in this meta-analysis. The following inclusion criteria were applied: (1) studies investigating risk factors for delirium occurrence following primary TJA; (2) inclusion of elderly individuals (≥60 years old) who underwent TJA; (3) definition of cases and controls based on the presence or absence of delirium, respectively; (4) utilization of Diagnostic and Statistical Manual of Mental Disorders IV edition criteria,^[11]^ or Diagnostic and Statistical Manual of Mental Disorders-derived criteria such as Confusion Assessment Method,^[12]^ for diagnosis; (5) availability of sufficient data for estimating odds ratios (ORs) or standardized mean differences (SMDs) with 95 % confidence interval (95 % CI).

### 2.3. Quality of included studies

The quality of the included studies was assessed using the Newcastle–Ottawa Scale,^[13]^ considering 3 main aspects: selection of study groups (0–4 points), comparability of groups (0–2 points), and ascertainment of either exposure or outcome of interest (0–3 points), with a maximum score of 9.

### 2.4. Data extraction

Data extraction was conducted independently by the 2 reviewers (Y.Z. and Y.Y.). Extracted variables from each study included the first author’s name, publication year, country, significant risk factors, definitions, numbers of cases and controls, and citations for each potential risk factor for delirium after total joint arthroplasty. Any discrepancies were resolved through discussion and consensus agreement.

### 2.5. Statistical analyses

ORs or SMDs and corresponding 95% CI were estimated and pooled across studies to assess the association between different variables and the risk of delirium (*P* < .05 considered significant). Heterogeneity among studies was assessed using Q-test statistics (*P* < .10 considered significant) and *I*^2^ statistics (*I*^2^ > 50% indicating significant inconsistency).^[14]^ A random-effects model was employed for pooled OR calculation in cases of significant heterogeneity; otherwise, a fixed-effects model was used. Meta-analysis outcomes were presented graphically using forest plots. Sensitivity analysis, excluding outlier studies one by one, was conducted to explore sources of heterogeneity if necessary. Potential publication bias was evaluated using Begg funnel plots, with *P* < .05 considered statistically significant. Statistical analyses were performed using Stata 11.0 software (Stata Corporation, College Station, TX).

### 2.6. Definitions of key terms

Demographic variables included age; gender; education; body mass index; current smoker; heavy drinker; mini-mental state examination (MMSE) score.

Physical status-related variables included co-morbidities, such as hypertension, diabetes mellitus, chronic kidney disease, history of stroke, coronary arterial disease, dementia, history of psychiatric illness (depression, anxiety disorders, schizophrenia, and others).

Surgery-related variables included American Society of Anesthesiologists rating scale, types of anesthesia; types of operation; operation time; anesthetic time; recovery time; blood transfusion (the treatment of allogeneic blood transfusion during or after surgery); intraoperative blood loss.

Drug-related variables included β-blockers, ACEIs, statins and psychotropic drug (antidepressants, anxiolytics, antipsychotics, sedatives, and hypnotics).

Laboratory variables: preoperative hemoglobin, preoperative white blood cell, preoperative C-reactive protein (CRP), preoperative albumin (ALB).

Postoperative complications included surgical site infection, dislocation, periprosthetic fracture, hematoma, and deep vein thrombosis.

## 3. Results

### 3.1. Characteristics of identified studies

**Figure 1.**
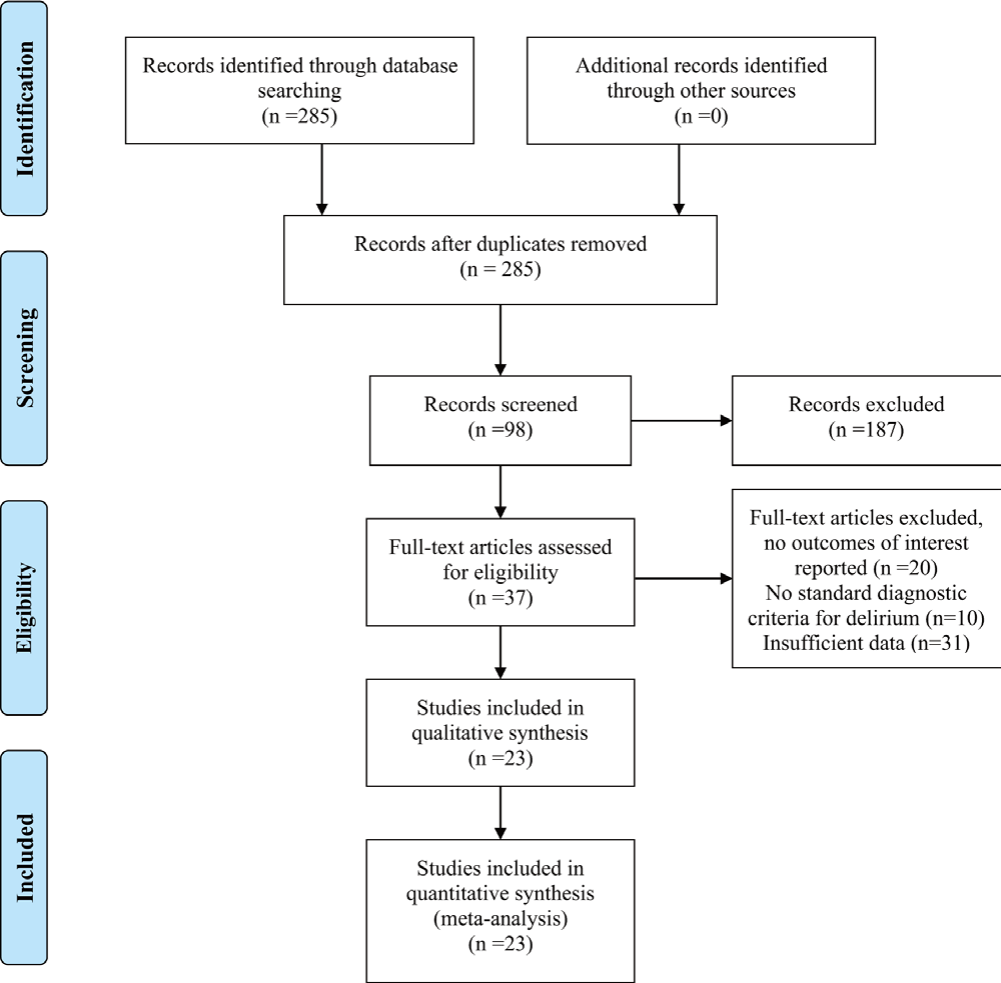
Flow chart of literature search.

Figure 1 displays the flowchart illustrating the screening process and subsequent selection steps. Initially, 285 titles and abstracts were retrieved from electronic databases. Following removal of duplicates, 98 abstracts underwent initial screening, with 37 progressing to the next stage of review. Subsequently, applying the inclusion and exclusion criteria resulted in the selection of 23 full-text articles for inclusion in the meta-analysis. All were published in English between 1995 and 2023. These 23 studies altogether included 71,095 patients, 2142 cases of delirium occurred after TJA, suggesting an accumulated incidence of 3.0%. Detailed information about these included studies is shown in Table 1.

**Table 1:** Detailed information on the basic characteristics of the 23 included studies and participants.

Author	Country	Publication year	Age (mean ± SD, year)		Sample size		Total	Rating scale	Significant factors	NOS score
			Delirium	Non-delirium	Delirium	Non-delirium				
Fisher	Canada	1995	71.2		14	66	80	CAM	Males	8
Freter	Canada	2005	76 ± .88		18	114	132	CAM	Substance use and cognitive impairment	8
Lowery	UK	2007	77.2 ± 4.3	76.3 ± 4.6	14	80	94	DSM-III	Preoperative attentional deficits	8
Priner	France	2008	78.2 ± 4.4	72.8 ± 6.6	15	86	101	CAM	Age, psychotropic drugs, knee surgery, blood loss intraoperatively	7
Jankowski	USA	2011	74.76 ± 6.07	72.74 ± 5.31	42	376	418	CAM	Age, history of psychiatric illness, decreased functional status, and decreased verbal memory	8
Bosmak	Brazil	2017	73.4	62.67	5	52	57	DSM-III	Females and hypertension	8
Petersen	Denmark	2017	80.7	76.68	43	746	789	DSM-IV	Age and length of stay > 4 days	7
Chen	China	2017	81.8 ± 4.9	72.2 ± 5.1	35	177	212	DSM-IV	Older age, a history of stroke, preoperative PaO_2_ and equivalent fentanyl dose	7
Cunningham	UK	2017	76.9 ± 6	74.0 ± 5.7	40	275	315	CAM	Time taken to complete colour trails 2, errors in mini mental state examination and level of pain preoperatively	8
Weinstein	USA	2018	78	66	922	40,844	41,766	DSM-IV	Anesthesia type and perioperative medications	9
Peng	China	2019	74.5 ± 5.6	72.1 ± 6.1	55	217	272	DSM-V	Age and preoperative C-Reactive protein/albumin ratio	8
Huang	USA	2019	75.4 ± 11.7	65.9 ± 12.2	181	11,789	11,970	CAM	Age, dementia, blood transfusions, and increased sedation during anesthetic recovery	8
Cunningham	UK	2019	76.6 ± 6.0	73.8 ± 5.6	40	242	282	CAM	Age, preoperative comorbidity, type of surgery, intravenous opioid usage and low CSF Aβ 42	9
Qi	China	2020	72.4 ± 4.1	72.1 ± 3.7	68	260	328	DSM-V	Serum albumin	8
Lin	China	2020	73.3 ± 6.5	72.2 ± 6.0	51	396	447	CAM	ChAT, AChE, BuChE	7
Meyer	Germany	2021	79 ± 7	66 ± 11	139	10,001	10,140	CAM	ASA classification	7
Chen	China	2021	72 ± 5		66	317	383	DSM-V	Age	8
Chen	China	2021	71.1 ± 9.6	66.4 ± 9.7	67	927	994	DSM-V	Prognostic nutritional index, age, hypertension	9
Chen	China	2021	85.06 ± 7.04	74.18 ± 6.05	65	195	260	CAM	Lactate dehydrogenase, cholinesterase, Cystatin C, arrhythmia and operation duration	8
Lin Xu	China	2022	77.38 ± 6.24	74.18 ± 6.05	61	271	332	CAM	Habitual tea consumption	7
Jiang	China	2022	74.3 ± 3.1	72.1 ± 2.9	43	293	336	DSM-V	Albumin/fibrinogen ratio	8
Lin Yanan	China	2022	68	61	66	496	562	CAM	TG, TC, LDL-C, HDL-C	7
Lin Yanan	China	2023	66.5	65	92	733	825	CAM	CSF Aβ 42, progranulin, α-syn, Aβ42/t-Tau and Aβ42/p-Tau	7

AChE = acetylcholinesterase, ASA = American Society of Anesthesiologists, BuChE = butyrylcholinesterase, CAM = Confusion Assessment Method, ChAT = plasma choline acetyltransferase, CSF = cerebrospinal fluid, DSM = Diagnostic and Statistical Manual of Mental Disorders, HDL-C = high-density lipoprotein, LDL-C = low-density lipoprotein, SD = standard deviation, TC = triglyceride, TG = total cholesterol.

### 3.2. Methodological quality assessment

The outcome of methodology quality assessment was as follows: 3 studies^[6,8,15]^ scored 9, 12 studies^[1,7,10,16–24]^ scored 8, and 8 studies^[25–32]^ scored 7.

### 3.3. Demographic factors

**Figure 2.**
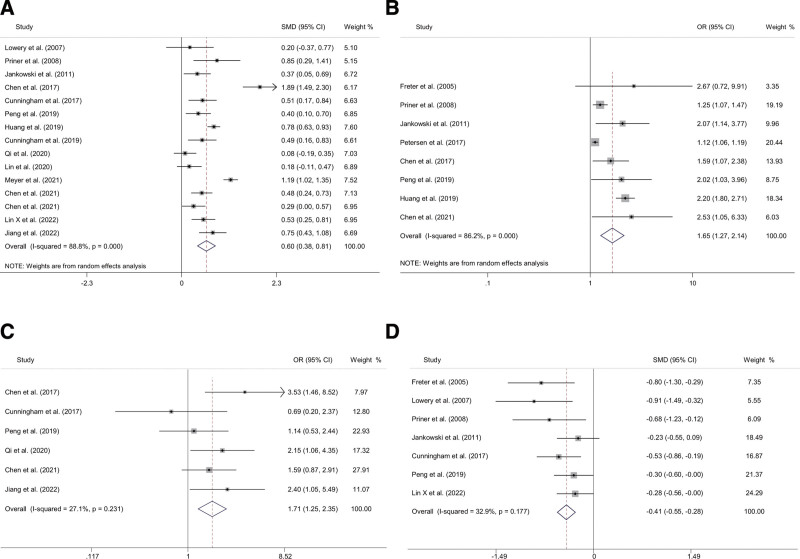
Forest plots of the meta-analyses of demographic factors comparing characteristics between delirium and non-delirium after primary TJA. Patients with the risk factors of (A) age (years), (B) age (high vs low), (C) heavy drinker, (D) MMSE score. TJA = total joint arthroplasty.

We performed a meta-analysis of 7 demographic factors that influenced delirium after primary TJA, including age, gender, body mass index, education level, current smoker, heavy drinker, and MMSE score (Table 2). Based on the statistical findings from the meta-analysis, the following factors were identified as influential (Fig. 2): age (years) (SMD 0.60, 95% CI 0.38–0.81), age (OR 1.65, 95% CI 1.27–2.14), heavy drinker (OR 1.71, 95% CI 1.25–2.35), MMSE score (SMD ‐0.41, 95% CI ‐0.55 to ‐0.28). The pooled analysis revealed that the risk of POD increased by 1.65 times for each year of age. Heavy drinker was the most serious risk factor for postoperative delirium after TJA, with a 1.71-fold increase in risk. In addition, A low MMSE score were risk factors for POD.

**Table 2:** Detailed data on potential risk factors for delirium after TJA and the outcomes of meta-analysis.

Potential risks	No of studies	Pooled OR or SMDs	LL 95% CI	UL 95% CI	*P*-value	Q-test for heterogeneity (*P*)	‡*I*² (%)
*Demographic factors*							
Age (years)	15	0.60	0.38	0.81	<.001†	<0.001	88.80
Age (high vs low)	8	1.65	1.27	2.14	<.001†	<0.001	86.20
Male	20	0.95	0.87	1.05	.318*	0.037	39.40
BMI (kg/m^2^)	8	0.04	‐0.13	0.21	.635†	0.003	68.10
Education (>high school vs ≤high school)	6	1.41	0.90	2.22	.137†	0.007	68.90
Current smoker	10	1.07	0.93	1.22	.359*	0.879	0.00
Heavy drinker	6	1.71	1.25	2.35	.001*	0.231	27.10
MMSE score	7	‐0.41	‐0.55	‐0.28	<.001*	0.177	32.90
*Physical status-related factors*							
Hypertension	8	1.23	1.01	1.50	.038*	0.391	5.10
Diabetes mellitus	9	1.38	1.07	1.78	.013*	0.037	51.20
Chronic kidney disease	4	1.82	1.41	2.36	<.001*	0.498	0.00
History of stroke	3	5.84	2.23	15.27	<.001†	0.047	67.30
Coronary arterial disease	5	1.62	1.17	2.26	.004*	0.176	36.80
Dementia	6	5.42	3.16	9.30	<.001*	0.056	53.70
History of psychiatric illness	4	3.62	2.58	5.08	<.001*	0.320	14.40
Sleep apnea	2	1.10	0.87	1.38	.434*	0.756	0.00
*Surgery-related factors*							
ASA physical status III–IV	8	1.51	1.12	2.04	.007*	0.032	54.20
Total hip arthroplasty	14	0.90	0.72	1.13	.348†	0.007	54.80
General anesthesia	4	1.80	1.45	2.23	<.001*	0.258	25.70
Anesthetic time (min)	5	0.22	0.10	0.34	<.001*	0.240	27.20
Anesthetic time (high vs low)	3	1.54	1.22	1.95	<.001*	0.722	0.00
Operative time (min)	9	0.20	0.06	0.34	.004*	0.005	63.40
Operative time (high vs low)	3	1.53	1.19	1.97	.001*	0.682	0.00
Recovery time (high vs low)	2	0.18	‐0.02	0.37	.084*	0.204	38.10
Intraoperative blood loss (ml)	4	0.23	0.08	0.38	.002*	0.923	0.00
Blood transfusion	6	1.84	1.37	2.46	<.001*	0.833	0.00
*Drug-related factors*							
β-blockers	3	1.82	1.22	2.70	.003*	0.661	0.00
ACEIs	3	1.61	1.01	2.56	.045*	0.775	0.00
Statins	2	1.01	0.53	1.94	.966*	0.712	0.00
Psychotropic drug use	2	2.52	1.29	4.92	.007*	0.608	0.00
*Laboratory factors*							
Preoperative hemoglobin (mg/dL)	3	‐0.10	‐0.27	0.08	.268*	0.636	0.00
Preoperative WBC (*10^9^/L)	2	0.02	‐0.18	0.22	.836*	0.304	5.20
Preoperative CRP (mg/L)	2	0.43	0.19	0.67	<.001*	0.410	0.00
Preoperative albumin (g/L)	4	‐0.35	‐0.59	‐0.12	.004†	0.053	60.90
*Postoperative complications*							
Surgical site infection	2	3.12	0.76	12.81	.115†	0.026	79.90
Dislocation	2	1.35	0.44	4.16	.603*	0.974	0.00
Periprosthetic fracture	2	2.12	0.55	8.25	.278*	0.450	0.00
Hematoma	2	1.38	0.55	3.47	.487*	0.693	0.00
Deep vein thrombosis	2	1.15	0.57	2.32	.699*	0.274	16.40

ASA = American Society of Anesthesiologists rating scale, BMI = body mass index, CRP = C-reactive protein, LL = lower limit, MMSE = mini-mental state examination, OR = odds ratio, UL = upper limit, WBC = white blood cell.

* Fixed-effects model was performed.

† Random-effects model was performed.

‡ *I*^2^ statistic was defined as the proportion of heterogeneity not due to chance or random error.

### 3.4. Physical status-related factors

**Figure 3.**
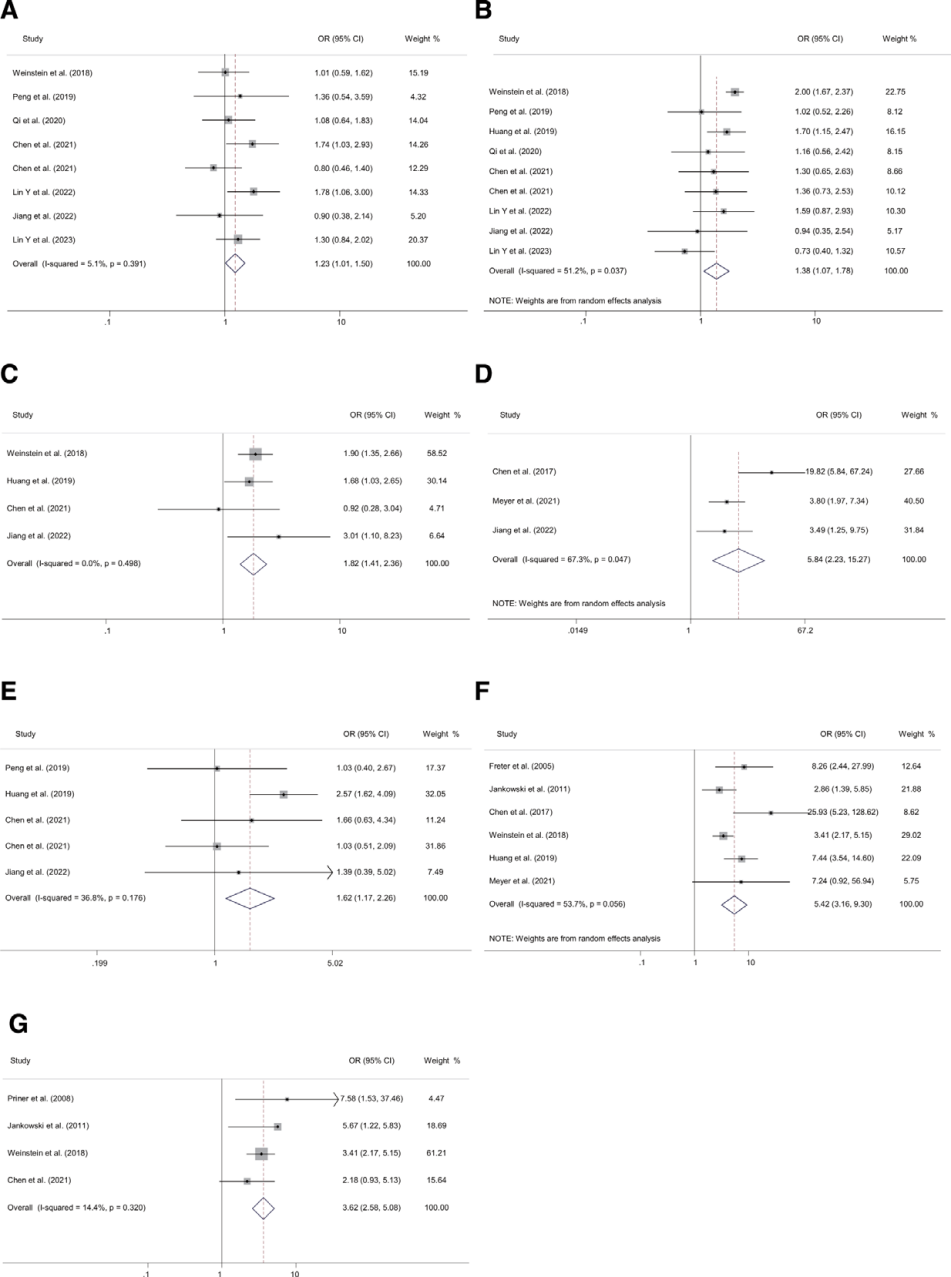
Forest plots of the meta-analyses of physical status-related factors comparing characteristics between delirium and non-delirium after primary TJA. Patients with the risk factors of (A) hypertension, (B) diabetes mellitus, (C) chronic kidney disease, (D) history of stroke, (E) coronary arterial disease, (F) dementia, (G) history of psychiatric illness. TJA = total joint arthroplasty.

We examined the impact of 8 comorbidities or medical history characteristics on postoperative delirium. The following risk factors were identified based on the pooled ORs (Fig. 3): hypertension (OR 1.23, 95% CI 1.01–1.50), diabetes mellitus (OR 1.38, 95% CI 1.07–1.78), chronic kidney disease (OR 1.82, 95% CI 1.41–2.36), history of stroke (OR 5.84, 95% CI 2.23–15.27), coronary arterial disease (OR 1.62, 95% CI 1.17–2.26), dementia (OR 5.42, 95% CI 3.16–9.30), history of psychiatric illness (OR 3.62, 95% CI 2.58–5.08). Sleep apnea was not a significant risk factor for POD after primary TJA.

### 3.5. Surgery-related factors

**Figure 4.**
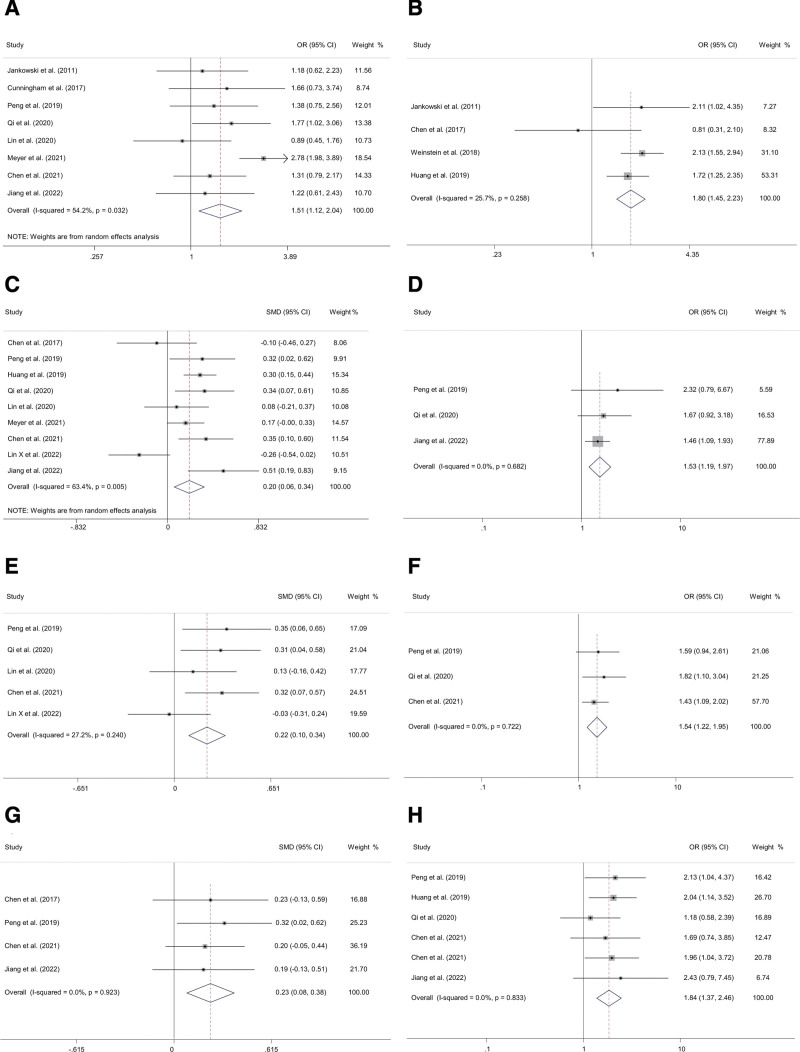
Forest plots of the meta-analyses of surgery-related factors comparing characteristics between delirium and non-delirium after primary TJA. Patients with the risk factors of (A) ASA physical status III–IV, (B) general anesthesia, (C) operative time (minutes), (D) operative time (high vs low), (E) anesthetic time (minutes), (F) anesthetic time (high vs low), (G) intraoperative blood loss (mL), (H) blood transfusion. TJA = total joint arthroplasty.

In this meta-analysis, American Society of Anesthesiologists (ASA) physical status III–IV (OR 1.51, 95% CI 1.12–2.04), general anesthesia (OR 1.80, 95% CI 1.45–2.23), anesthetic time (minutes) (SMD 0.22, 95% CI 0.10–0.34), operative time (minutes) (SMD 0.20, 95% CI 0.06–0.34), intraoperative blood loss (mL) (SMD 0.23, 95% CI 0.08–0.38), blood transfusion (OR 1.84, 95% CI 1.37–2.46) were significantly related to delirium after primary TJA (Fig. 4). Notably, we did not find any significant difference in the incidence of POD between hip and knee arthroplasty patients.

### 3.6. Drug-related factors

**Figure 5.**
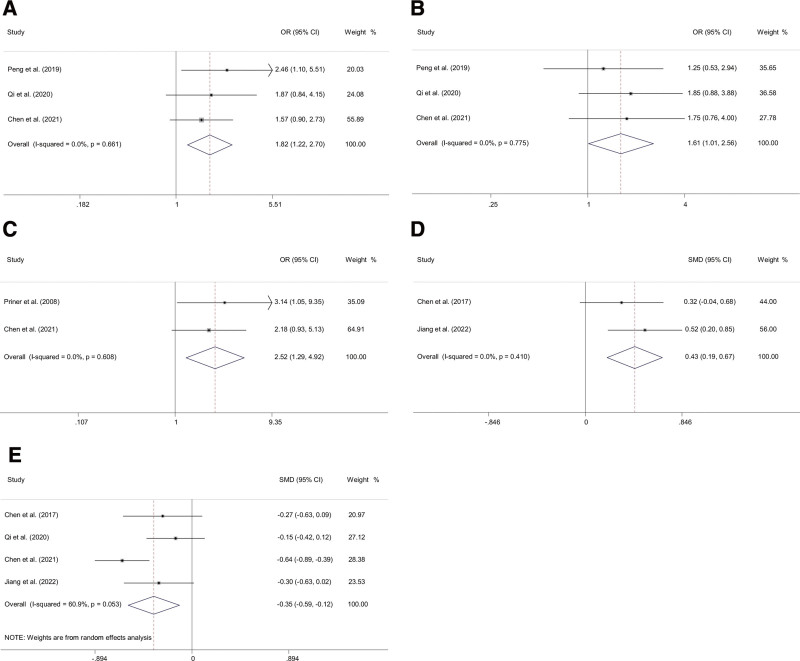
Forest plots of the meta-analyses of drug-related factors and laboratory factors comparing characteristics between delirium and non-delirium after primary TJA. Patients with the risk factors of (A) β-blockers, (B) ACEIs, (C) psychotropic drug use, (D) preoperative CRP (mg/L), (E) preoperative albumin (g/L). ALB = albumin, CRP = C-reactive protein, TJA = total joint arthroplasty.

In drug-related factors, β-blockers (OR 1.82, 95% CI 1.22–2.70), ACEIs (OR 1.61, 95% CI 1.01–2.56), use of psychotropic drug (OR 2.52, 95% CI 1.29–4.92) indicated a significantly increased risk of POD (Fig. 5). The use of statins was not a significant risk factor for POD.

### 3.7. Laboratory factors

The pooled analysis of the included studies revealed that patients with higher preoperative CRP (SMD 0.43, 95% CI 0.19–0.67) or lower preoperative ALB levels (SMD ‐0.35, 95% CI ‐0.59 to ‐0.12) were more prone to develop delirium following TJA. However, the association of preoperative levels of hemoglobin and white blood cell with POD in patients undergoing TJA was not significant (Fig. 5).

### 3.8. Postoperative complications

There were 2 included studies reporting the postoperative complications after TJA. However, patients with surgical site infection, dislocation, periprosthetic fracture, hematoma and DVT did not show a higher incidence of POD than those without.

### 3.9. Publication bias

**Figure 6.**
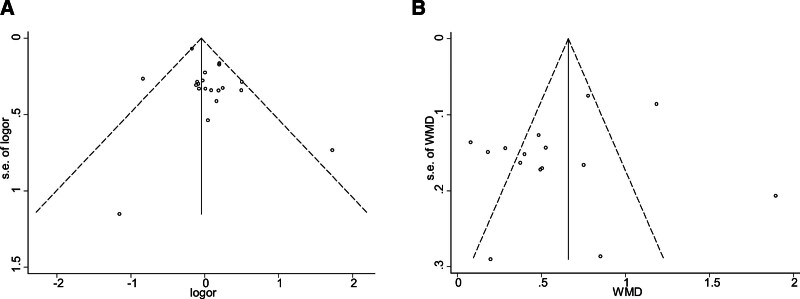
Begg funnel plot for publication bias (with 95% pseudo confidence limits) of the observational studies that investigated male gender and age differences between delirium and non-delirium after primary TJA. TJA = total joint arthroplasty.

The funnel plots for male gender were examined visually and its shape was basically symmetrical. Examination for publication bias via Begg test indicated the absence of potential publication bias within the studies included in the analysis (*P* = .103; Fig. 6).

### 3.10. Sensitivity analysis

In this meta-analysis, 8 risk factors showed significant heterogeneity (*P* < .10 or *I*^2^ > 50%) and could be further analyzed. This meta-analysis found a significant heterogeneity for the age factor (age (years): *P* < .001, *I*^2^ = 88.8%; age (high vs low): *P* < .001, *I*^2^ = 86.2%). However, after excluding outlier studies in a sensitivity analysis, the *I*^2^ value decreased to 38.0% and 35.0%, respectively, and the significance did not change, indicating the reliability of the result (Table S1, Supplemental Digital Content, http://links.lww.com/MD/M748). The heterogeneity in age may be attributed the data recall bias resulting from retrospective studies and the large age gap between the delirium and non-delirium groups. The other risk factors (i.e., diabetes mellitus, history of stroke, dementia, ASA physical status III–IV, operative time [minutes], and preoperative ALB [g/L]) also showed a lower heterogeneity after removing one article, and the significance was consistent. The results of the sensitivity analysis were presented in Table S1, Supplemental Digital Content, http://links.lww.com/MD/M748.

## 4. Discussion

This meta-analysis aimed to identify the risk factors for POD after primary TJA of the hip and knee. We analyzed 6 categories of risk factors based on 23 studies involving 71,095 patients, who had a POD incidence of 3.0% after primary TJA. The results indicated that age, current smoker, heavy drinker, MMSE score, hypertension, diabetes mellitus, chronic kidney disease, history of stroke, coronary arterial disease, dementia, history of psychiatric illness, ASA physical status III–IV, general anesthesia, anesthesia time, operative time, intraoperative blood loss, blood transfusion, β-blockers, ACEI drugs, use of psychotropic drugs, preoperative CRP level, and preoperative ALB level were significantly associated with postoperative delirium after primary TJA.

### 4.1. Demographic factors

Consistent with previous studies, we found that age was a significant risk factor for POD after primary TJA.^[5]^ Age may reflect frailty and cognitive reserve, which affect the susceptibility and resilience to delirium. Furthermore, older patients may experience an elevated POD incidence due to heightened comorbidities, age-associated alterations in organ and brain architecture, pharmacodynamics, renal function, and metabolism.^[33]^ Additionally, our investigation identified heavy drinking as a significant risk factor for POD subsequent to primary TJA. This association may be linked to the manifestation of alcohol withdrawal syndrome symptoms following surgery, particularly among patients with a history of substantial alcohol consumption.^[34]^ Moreover, we observed that MMSE score served as a significant protective factor against postoperative delirium (POD) following primary TJA. This metric reflects patients’ baseline cognitive function, which shapes their capacity to manage perioperative stress and adjustments. Several investigations^[35,36]^ have demonstrated the feasibility of preoperative cognitive testing in emergency situations. Thus, its incorporation into the standardized preoperative clinical assessment protocol for surgeries involving TJA is warranted.

### 4.2. Physical status-related factors

We found that several co-morbidities were significant risk factors for POD after primary TJA, such as hypertension, diabetes mellitus, chronic kidney disease, history of stroke, coronary arterial disease, dementia, and history of psychiatric illness. The etiology of delirium in patients with diabetes mellitus, poststroke, dementia, or psychiatric illness was unclear, but there was consensus on the association between these conditions and delirium.^[27]^ These co-morbidities may affect the cerebral perfusion and metabolism,^[37]^ increase the neuroinflammation and oxidative stress,^[38,39]^ and disrupt the neurotransmitter regulation.^[40]^ These factors may predispose the brain to delirium in response to perioperative stressors.^[28]^ Lin et al found that lower levels of cerebrospinal fluid (CSF) cholinergic markers were significantly correlated with higher POD risk in elderly patients undergoing TJA.^[28]^ Similarly, chronic kidney disease introduces complex physiological changes, including electrolyte imbalances, uremic toxins, and neurohormonal dysregulation.^[41]^ These factors can disrupt neuronal functioning and neurotransmitter regulation, potentially contributing to the onset of delirium. Our findings align with prior research, highlighting hypertension, and coronary artery disease as additional risk factors for the development of postoperative delirium.^[42]^ Clinicians should consider these factors and provide appropriate risk stratification and individualized perioperative care to minimize delirium occurrence and impact.

### 4.3. Surgery-related factors

Surgery-related factors emerged as significant contributors to the development of POD after primary TJA. ASA physical status III–IV, which reflects the overall health status and functional reserve of the patients,^[29]^ was a significant risk factor for POD after primary TJA. It may influence their tolerance and recovery from surgery. When contrasted with regional anesthesia, general anesthesia emerged as a notable risk factor for POD following primary TJA. This heightened risk may be attributed to its potential to escalate opioid utilization, surgical stress, inflammatory reactions, and the demand for general anesthetics during major surgery.^[43,44]^ Anesthesia time and operative time were also significant risk factors for POD after primary TJA. Extended anesthesia and operative periods may heighten patient exposure to diverse anesthetic agents and surgical stressors, potentially compromising cerebral perfusion and metabolism, accentuating neuroinflammation and oxidative stress, and perturbing neurotransmitter equilibrium.^[44]^ Furthermore, this meta-analysis ascertained that intraoperative blood loss and blood transfusion were significant risk factors for POD after primary TJA. Intraoperative blood loss may cause hypotension and hypoxia, which may reduce the cerebral oxygen delivery and increase the risk of ischemic brain injury. Ou Yang et al^[9]^ proposed that allogeneic blood transfusion could trigger an inflammatory response and oxidative damage in the patient, which might explain the pathophysiological link between blood transfusion and POD.

### 4.4. Drug-related factors

The present study unveiled a range of drugs as noteworthy risk contributors to POD following primary TJA, including β-blockers, ACEI drugs, and psychotropic agents. Specifically, β-blockers and ACEI drugs were discerned for their potential to induce reductions in blood pressure and cerebral perfusion pressure, potentially fostering an environment conducive to heightened ischemic brain injury and amplified risk of delirium.^[45]^ Similarly, the administration of psychotropic drugs may introduce sedative, anticholinergic, or dopaminergic effects, potentially leading to compromises in levels of consciousness, cognitive function, and neurotransmitter equilibrium.^[46,47]^

### 4.5. Laboratory factors

This meta-analysis revealed that preoperative CRP and ALB levels emerged as noteworthy risk factors for POD following primary TJA. Preoperative CRP level reflects the systemic inflammation status of the patients, which may affect the neuroinflammation and blood–brain barrier permeability.^[24]^ Similarly, the preoperative ALB level is indicative of the patients’ nutritional status, a variable with potential repercussions on immune function.^[21,24]^ These factors may predispose the brain to delirium in response to perioperative stressors. CSF, being the bodily fluid most directly indicative of nervous system changes, holds significant diagnostic, and predictive value for POD.^[6,28]^ However, the scarcity of relevant studies precluded our ability to discern the impact of CSF-related markers on the occurrence of POD.

### 4.6. Postoperative complications

We did not find any significant association between postoperative complications and POD after primary TJA. This may be due to the heterogeneity of how postoperative complications were defined and measured among the included studies. Furthermore, postoperative complications may result from rather than cause delirium. More studies are needed to clarify the causal relationship between postoperative complications and POD after primary TJA.

## 5. Limitations

This meta-analysis has some limitations. First, we only searched English-language databases, which may have missed some relevant studies in other languages. Second, we only analyzed the risk factors that were reported by at least 3 studies, which may have overlooked some important but less studied risk factors. Third, we found significant heterogeneity among the included studies, which may have affected the accuracy and generalizability of our results.

## 6. Conclusion

In conclusion, our meta-analysis identified several risk factors for POD after primary TJA of the hip and knee. These risk factors can be used to screen and stratify the patients who are at high risk of developing delirium after surgery. Moreover, these risk factors can be used to guide the prevention and management of delirium by modifying or avoiding the modifiable risk factors and optimizing or treating the non-modifiable risk factors. Further large-sample, high-quality, and well-documented prospective studies are necessary to support these findings.

## Acknowledgments

We are grateful to Z. Y. and G. L. of the Department of Orthopedics, and to W. J. and Z. L. of the Department of Statistics and Applications for their kind assistance.

## Author contributions

**Conceptualization:** Yanju Zhang, Yanjie Yu.

**Data curation:** Ziyu Han, Yanjiang Yang.

**Investigation:** Ziyu Han, Runping Zhao.

**Resources:** Li Diao.

**Software:** Jinzhu Liu.

**Supervision:** Yuhong Luo.

**Visualization:** Huiyuan Wu.

**Writing – review & editing:** Yanjiang Yang.

## Supplementary Material


