# Supplementary material for: Incidence and associated factors of delirium after primary total joint arthroplasty in elderly patients: A systematic review and meta-analysis
Source: Medicine (Baltimore). 2024 May 31;103(22):e38395. doi: 10.1097/MD.0000000000038395 (PMC11142822; doi:10.1097/MD.0000000000038395)
Supplement: Supplementary file 1 [file medi-103-e38395-s001.doc]

Supplementary table 1 Results of sensitive analysis for variables

| Variables | OR/SMD and corresponding 95%CI (original) | P for heterogeneity | I^2^ | The outlier study excluded | OR and corresponding 95%CI (afterwards) | P for heterogeneity | I^2^ |
| --- | --- | --- | --- | --- | --- | --- | --- |
| Age (years) | 0.60 (0.38-0.81) | 0.001 | 88.8% | Chen 2017  Huang 2019  Meyer 2021 | 0.40 (0.31-0.49) | 0.088 | 38.0% |
| Age (high vs low) | 1.65 (1.27-2.14) | 0.001 | 86.2% | Petersen 2017  Huang 2019 | 1.38 (1.20-1.59) | 0.174 | 35.0% |
| Diabetes mellitus | 1.38 (1.07-1.78) | 0.037 | 51.2% | Lin Y 2023 | 1.76 (1.53-2.02) | 0.296 | 17.0% |
| History of stroke | 5.84 (2.23-15.27) | 0.001 | 67.3% | Chen 2017 | 3.69 (2.11-6.46) | 0.892 | 0 |
| Dementia | 5.42 (3.16-9.30) | 0.056 | 53.7% | Chen 2017 | 4.14 (3.03-5.67) | 0.203 | 32.7% |
| ASA physical status III–IV | 1.51 (1.12-2.04) | 0.032 | 54.2% | Lin 2020 | 1.77 (1.45-2.16) | 0.072 | 48.2% |
| Operative time (min) | 0.20 (0.06-0.34) | 0.005 | 63.4% | Lin X 2022 | 0.26 (0.17-0.34) | 0.196 | 29.1% |
| Preoperative albumin (g/L) | -0.35 (-0.59--0.12) | 0.053 | 60.9% | Qi 2020 | -0.46 (-0.63--0.29) | 0.132 | 50.5% |
